# Selecting among Alternative Scenarios of Human Evolution by Simulated Genetic Gradients

**DOI:** 10.3390/genes9100506

**Published:** 2018-10-18

**Authors:** Catarina Branco, Miguel Arenas

**Affiliations:** Department of Biochemistry, Genetics and Immunology, University of Vigo, 36310 Vigo, Spain; caraujo@uvigo.es

**Keywords:** human genetic gradients, human evolution, model selection, range expansion, range contraction, last glacial maximum, long-distance dispersal, allele surfing

## Abstract

Selecting among alternative scenarios of human evolution is nowadays a common methodology to investigate the history of our species. This strategy is usually based on computer simulations of genetic data under different evolutionary scenarios, followed by a fitting of the simulated data with the real data. A recent trend in the investigation of ancestral evolutionary processes of modern humans is the application of genetic gradients as a measure of fitting, since evolutionary processes such as range expansions, range contractions, and population admixture (among others) can lead to different genetic gradients. In addition, this strategy allows the analysis of the genetic causes of the observed genetic gradients. Here, we review recent findings on the selection among alternative scenarios of human evolution based on simulated genetic gradients, including pros and cons. First, we describe common methodologies to simulate genetic gradients and apply them to select among alternative scenarios of human evolution. Next, we review previous studies on the influence of range expansions, population admixture, last glacial period, and migration with long-distance dispersal on genetic gradients for some regions of the world. Finally, we discuss this analytical approach, including technical limitations, required improvements, and advice. Although here we focus on human evolution, this approach could be extended to study other species.

## 1. Introduction

The evolutionary history of our species persists as a hot topic of research due to the curiosity about our past and the continuous interesting findings from both genetic and archeological data, despite the fact that these findings are sometimes contradictory e.g., [1,2,3]. Indeed, knowledge about human genetic variation may help us to understand the causes and effects of some human diseases, like those presenting variable behaviour among ethnic groups or populations e.g., [4,5]. Conveniently, the genetic material of current humans still presents signatures of past evolutionary events, allowing us to investigate aspects about our origins. However, the interpretation of these findings is not always straightforward, because different evolutionary processes can lead to similar results. A clear example is the interpretation of the genetic gradients (clines) of modern humans by Cavalli-Sforza et al. [6,7,8]. These gradients were initially explained as genetic signatures of specific migrations. For example, Cavalli-Sforza et al. interpreted the European southeast–northwest (SE-NW) gradient of genetic variation as the result of the demic diffusion of early Neolithic farmers during their expansion from the Near East [9,10]. Posterior studies suggested that such genetic gradients could be caused or influenced by other processes such as range contractions or population admixture, i.e., hence, not necessarily attributed to a particular range expansion [11,12,13,14,15]. Interestingly, applying spatially-explicit computer simulations, François et al. [12] and Arenas et al. [14] showed that genetic gradients can present a direction perpendicular to the direction of the expansion as a consequence of *allele surfing* [16,17], where mutations occurring on the wave of advance of the range expansion generate highly-differentiated genetic sectors aligned perpendicular to the direction of the expansion. Allele surfing is more detectable in recent expansions of small populations and under low migration rates, where sectors were not yet removed through homogenization [17]. Genetic gradients can also present the direction of the range expansion if the genetic signatures of allele surfing are lower than the genetic signatures of other genetic processes, such as isolation by distance (IBD). For example, Branco et al. [18] recently studied the influence of different evolutionary scenarios on American genetic gradients of modern humans through extensive spatially-explicit computer simulations. They found that at the continental level, the genetic gradient presented a direction following that of the range expansion under any studied evolutionary scenario (see Section 3), which was explained as IBD (similarly to the interpretations by Cavalli-Sforza et al. [7]), but this gradient varied when studied in smaller geographic regions, suggesting that the influence of different genetic processes on genetic gradients can vary with the geographic features of the landscape.

Since recent studies showed that genetic gradients can vary with different evolutionary processes, one can perform a selection among alternative evolutionary scenarios with data simulated under each scenario, followed by a fitting between simulated and real data based on genetic gradients. This strategy is not new in population genetics; for instance, the approximate Bayesian computation (ABC) approach [19,20] is frequently used to evaluate alternative scenarios of human evolution e.g., [21,22,23,24,25]. A goal of ABC is that it provides a quantitative evaluation of the fitting between real and simulated data; however, on the other hand, it usually requires a huge number of computer simulations (from many thousands to millions, although they can run in parallel) to obtain results with an acceptable level of accuracy and precision. Concerning the analyses based on a comparison between real and simulated genetic gradients, the most recent studies only required hundreds of simulations to identify the best fitting scenario, but these comparisons were mainly qualitative (direction of genetic gradients).

Here, we review the application of genetic gradients simulated under spatially-explicit computer simulations to distinguish between alternative evolutionary scenarios of modern humans by their fitting with real genetic gradients. First, we present the commonly-used methodologies to perform this selection among alternative scenarios, including the simulation of genetic data and estimation of genetic gradients. Next, we describe previous studies applying this approach to investigate the influence of human range expansions, range contractions followed by range re-expansions (processes that can be induced by glacial periods), population admixture and migration with long-distance dispersal, among others, on genetic gradients of some regions of the world, and to perform a selection among alternative evolutionary scenarios. Finally, we discuss advantages and limitations of those studies, and we provide recommendations based on our experience.

## 2. Simulation of Genetic Gradients

The simulation of genetic gradients usually consists of two main steps, namely: the simulation of genetic data under a given evolutionary scenario, and the estimation of the genetic gradient from the simulated data. Next, we describe the most frequently-used methodologies to perform both steps.

### 2.1. Simulation of Genetic Data under Diverse Evolutionary Scenarios of Human Evolution

A variety of approaches exist to simulate genetic data in population genetics, and they can be roughly classified in two types concerning the kind of simulation: (*i*) simulation of the evolutionary history of a sample, and (*ii*) simulation of genetic data upon a given evolutionary history.

Concerning the simulation of the evolutionary history of a sample, a number of approaches have been developed. The most commonly-used approaches are the coalescent [26], the birth-death approach [27], and the forward-time approach [28]. Basically, the coalescent simulates the evolutionary history of a sample of alleles from the present to the past until their most recent common ancestor (MRCA). The birth-death approach simulates the evolution of a sample considering birth and death rates, which drive the amount of variability (branching) in the simulated history. By contrast, the forward-time approach simulates the evolution of a whole population from the past to the present. Despite the fact that the forward-time approach incorporates more evolutionary processes than the other approaches (i.e., interactions among individuals e.g., [29], population admixture e.g., [30], complex selection e.g., [29,31], and complex migration models e.g., [30,32,33]), computer simulations performed under this approach are computationally-intensive because of the simulation of many individuals (although progress is being made in this respect e.g., [34]). The simulation of the evolutionary history under a birth-death approach is much faster (similarly to the coalescent) but requires prior knowledge about birth and death rates. The coalescent is possibly the most commonly-implemented approach to be applied in population genetics (including studies on human evolution e.g., [23,35]), probably because of its rapid computation, its similarity with population genetics processes by modeling evolution based on the population size, and because it is capable of taking into account additional evolutionary processes such as demographics [36], recombination [37,38], population structure and migration [39,40,41], or selection e.g., [42,43,44,45]. Indeed, because of its rapid simulation and realistic population genetics modeling, the coalescent is a very useful approach when extensive simulations are required, for example in studies based on ABC or Bayesian approaches. For further details about approaches and frameworks to simulate evolutionary histories, we recommend the following reviews [46,47,48]. Interestingly, the forward-time and coalescent approaches were combined into the simulator *SPLATCHE*, allowing a rapid simulation of the evolutionary history of a sample accounting for evolutionary processes acting at the whole population level [49,50]. Basically, this framework simulates a spatial and temporal evolution of the whole population followed by the reconstruction of the evolutionary history of a given sample that is embedded in the previously-simulated population [50]; further details are shown later. This technical innovation made this framework well established in population genetics studies of terrestrial species, including humans [51].

Once the evolutionary history of the sample is simulated (i.e., a simulated phylogenetic tree), it is possible to simulate molecular evolution upon such evolutionary history to obtain genetic sequences for all the internal and tip nodes (note that the set of simulated sequences of the tip nodes can compose a multiple sequence alignment) [46,52,53]. The traditional procedure is based on the following two steps: First, a genetic sequence (random or devised by the researcher) must be assigned to the MRCA node. Second, the MRCA sequence is evolved, from the past to the present, over the evolutionary history to obtain a sequence for every internal and tip (sample) node (an illustrative example is presented in the following subsection). The number of simulated substitutions depends on the branch length, while the type of simulated substitutions depends on the specified substitution model of evolution [46,52,54].

#### Spatially-Explicit Computer Simulations

It is known that the consideration of a 2-dimensional (2D) landscape with its particular geography may result in simulations which are more realistic than those obtained with models of a lower number of dimensions [28,55]. This is because the real processes are often influenced by spatial constraints that may also vary over time, leading to the need for spatially-explicit models of evolution [56,57]. Despite some computer simulators implementing spatially-explicit models [49,50,58,59,60], unfortunately, several of them are not available to the public (i.e., the tool developed by Rendine et al. [61] applied to simulate an European Paleolithic and Neolithic expansion with admixture and the tool developed by Rasteiro et al. [30] applied to simulate human sex-biased migration). Other spatially-explicit computer simulators (i.e., *KERNELPOP* [59], *IBDSim* [60] and *CDMetaPOP* [62]) have not been yet widely applied to the study of human evolution, but they are potentially applicable for that purpose (see [28,51] for comparisons among different Spatially-explicit computer simulators). Next, the spatially-explicit computer simulator *SPLATCHE* [50] and its second version *SPLATCHE2* [49] have been largely used to study human evolution, perhaps because of their variety of implemented capabilities and their graphical user interface. Hence, hereafter we focus on this simulator, which is the simulator used in the studies presented in the following sections of this review.

**Figure 1:**
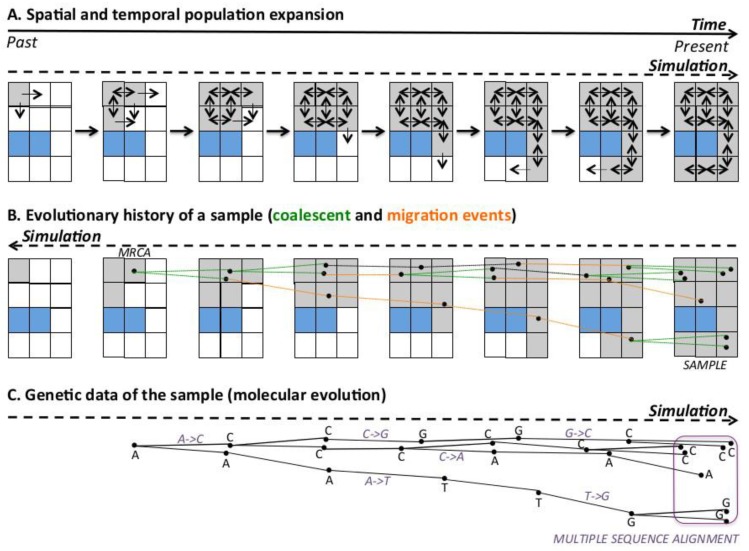
Illustrative example of a spatially-explicit simulation of a range expansion according to a 2-dimensional (2D) stepping-stone migration model [64], followed by the reconstruction of the evolutionary history of the sample and the simulation of genetic data. (**A**): Population range expansion, from the past to the present. It starts from the upper-left deme (origin), and migrants are sent to neighboring demes. Colonized demes (gray) can send/receive individuals to/from the neighboring demes, while non-colonized demes (white) can only receive individuals. We included a region representing a sea that cannot be colonized (blue), constituting a spatial barrier to migration. (**B**): Reconstruction of the evolutionary history of a sample of 7 individuals (present). Going backwards in time, coalescence (green) and migration (orange) events occur until the most recent common ancestor (MRCA) of the sample is reached, which does not necessarily correspond to the origin (time and place) of the range expansion. (**C**): Simulation of genetic data for the sample. A random sequence (for simplicity, in this example, it is just 1 nucleotide, (**A**)) is evolved forward in time, incorporating substitutions along branches (violet), until reaching the sample (present). At the end of the simulation, a multiple sequence alignment is obtained by combining all the sequences of the sample. Note that the spatial barrier can affect the shape of the evolutionary history of the sample, and consequently, the genetic information of the sample.

**Figure 2:**
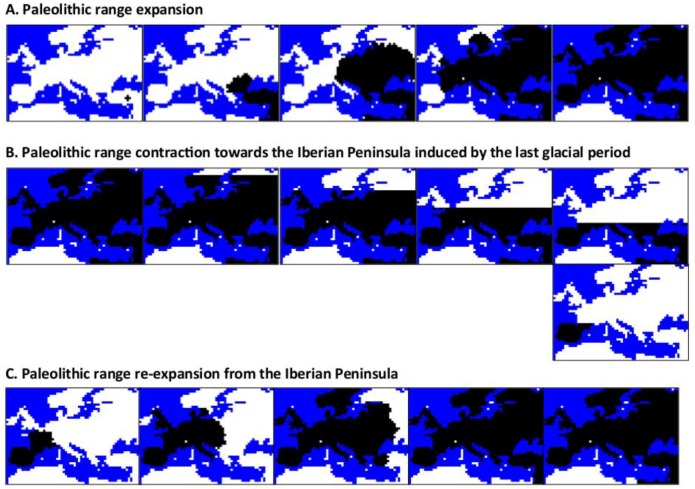
Illustrative example of the simulation of spatial and temporal expansion, contraction, and re-expansion of Paleolithic Europeans. The figure presents snapshots obtained with the program *SPLATCHE2* for an example of: (**A**) simulation of a Paleolithic range expansion over Europe, (**B**) simulation of a Paleolithic range contraction towards the Iberian Peninsula induced by the last glacial maximum (LGM), and (**C**) simulation of a Paleolithic range re-expansion from the Iberian Peninsula after the LGM. To perform this simulation, we applied settings similar to those specified in [14]. Note that the time moves from the left to the right and the range expansion starts from the bottom-right corner of Europe (Middle East). Snapshots are taken each 50 generations. White demes indicate empty regions and black demes indicate colonized regions. Note that after this Paleolithic expansion, contraction and re-expansion, a Neolithic expansion (also from the Middle East) could be simulated with or without admixture with Paleolithic populations.

Spatially-explicit computer simulations with *SPLATCHE2* require a 2D landscape/map, which, for various regions of the world, can be imported from a Geographical Information System (GIS) [63]. This map can be split into a grid of small areas (demes) with a given deme size. *SPLATCHE2* simulates samples of genetic data by three main steps (Figure 1): (*i*) A forward-in-time simulation of the evolutionary history of the entire population accounting for spatial and demographic information (Figure 1A). Here, a deme must be chosen as a point of origin to start an expansion over the space and time. Next, migration events occur towards neighboring demes under the 2D stepping-stone migration model [64]. Each deme can be modeled with particular environmental conditions such as a particular carrying capacity (a measure of the resources available in the deme) and friction (capacity to move through the deme), and these parameters can vary over time to mimic periods with different resources. Next, the variation of the population size over time for each deme depends on the population growth rate and the specific environmental parameters of the deme. Indeed, the number of migration events from each deme depends on the migration rate and population size of the deme [50]. The simulation occurs during a user-specified number of generations that should be higher than the time to the MRCA (TMRCA) of the sample. An illustrative example of simulation of spatial and temporal expansion of European modern humans is shown in the Figure 2. The next steps consist of: (*ii*) the application of the coalescent to reconstruct the evolutionary history of a sample (which is embedded in the history of the entire population) (Figure 1B) and, (*iii*) a simulation of molecular (sequence) evolution over the evolutionary history of the sample to obtain genetic data for the sample (Figure 1C). *SPLATCHE2* can simulate genetic sequences with diverse molecular markers, including DNA, single nucleotide polymorphism (SNP), and short tandem repeat (STR).

### 2.2. Estimation of Genetic Gradients in Studies of Human Evolution

Nowadays, several approaches allow the estimation of a genetic gradient from a dataset of genetic sequences. The traditionally-applied method to estimate genetic gradients is the principal component analysis (PCA). PCA identifies orthogonal axes (principal components, PCs) where objects show the highest variance of the information present in the original data. In population genetics, PCs provide an acceptable approximation of the covariance pattern among individuals of a given dataset [12]. They were largely used to study human evolution by Cavalli-Sforza [65], with the estimation of genetic gradients of European populations from allele-frequency data, and posteriorly used to estimate genetic gradients for other worldwide human populations [6,7]. Nowadays, PCA remains a very useful and powerful technique to estimate genetic gradients [11,66] because it properly summarizes information present in large genetic data [67,68]. Recent studies that used PCA to obtain genetic gradients [12,14,18] applied the “*prcomp*” function of the *R* software environment. Indeed, the studies by Arenas et al. [14] and Branco et al. [18], which performed a high number of computer simulations per analyzed evolutionary scenario, estimated a genetic gradient for each simulated dataset. Next, they connected the geographical centroids of the positive and negative coordinates for every gradient to obtain a line representing the direction of the gradient. Finally, in order to summarize all the simulated genetic gradients obtained from each evolutionary scenario, they computed the median of the lines (slope and intercept) of the simulated gradients per scenario.

Recently, more complex methods have been developed to estimate genetic gradients and population structures for a given landscape [69,70,71]. These methods apply the Bayesian approach to infer genetic variation by modeling genetic distances between populations as a function of their geographic distance e.g., [72,73,74]. A limitation of these methodologies is that they may require long computer times to obtain convergence among MCMC chains, and, from our experience (unpublished), can generate artifacts when inferring genetic gradients in non-sampled regions (extrapolation of a genetic gradient). Rapid estimation with PCA is convenient for studies based on a high number of genetic datasets, like those presented in the following sections of this review involving many computer simulations. We believe that comprehensive comparisons of performance among the new Bayesian methods, and also including the PCA methods, should be investigated (for example by computer simulations).

## 3. Selecting among Alternative Scenarios of Population Admixture through Simulated Genetic Gradients

The European settlement by Paleolithic and Neolithic populations has been generally proposed with little admixture, yet studies still disagree. The estimated level of admixture varied with the applied genetic marker and the type of analyses performed, with Neolithic contributions below 25% [75], near 50% [76], and above 50% [9,77,78]. Assuming that the level of admixture could affect genetic gradients, a few studies investigated the amount of admixture by fitting genetic gradients simulated under different levels of admixture with the observed (real) genetic gradients obtained by Cavalli-Sforza et al. [6,7]. The gradients found by Cavalli-Sforza et al. present a SE-NW orientation, and were originally interpreted by these authors as a consequence of a demic diffusion process of Neolithic farmers from the Near East [6,7,8,79]. A first study exploring the influence of Paleolithic-Neolithic admixture through spatially-explicit computer simulations was performed by Currat and Excoffier [78]. They always found a gradient with a direction following the Neolithic expansion (SE-NW). Later, François et al. [12] repeated the study analyzing several levels of admixture. Under a high proportion (>20%) of Neolithic ancestry, they found a genetic gradient with a SW-NE direction, which is perpendicular to the direction of the range expansion from the Middle East. This gradient was interpreted as a consequence of allele surfing (see Introduction). Under lower levels of Neolithic ancestry, the gradient presented a direction following the direction of the range expansion (fitting with the gradient obtained from real data by Cavalli-Sforza et al.); this gradient was interpreted as a Paleolithic introgression along the direction of the Neolithic expansion. However, François et al. [12] only performed 10 simulations per studied evolutionary scenario and ignored some evolutionary processes such as range contractions induced by the last glacial maximum (LGM) period (Section 4). In a posterior study, Arenas et al. [14] repeated the analyses with more sophisticated evolutionary scenarios (including a variety of levels of Paleolithic-Neolithic admixture and range contractions modeling the effect of the LGM, as discussed in Section 4), and increased to 100 the number of simulations per studied evolutionary scenario. They verified that under high levels of Neolithic ancestry (>20%), the genetic gradients follow a direction perpendicular to the direction of the range expansion (allele surfing). By contrast, under low levels of Neolithic ancestry, the genetic gradient followed the direction of the range expansion. However, they also found that the LGM could also affect the genetic gradients, leading to a more complex system that we present in Section 4.

The genetic gradient in the Americas based on real data follows the direction of the expansion (NW-SE) from Bering [6,7,80]. A similar gradient was also obtained from the analysis of the geographic distribution of linguistic families and subfamilies in this continent [7]. Recently, we and coauthors tried to investigate the level of admixture between the first Amerindian populations by applying computer simulations spatially [18]. We simulated two hypothetical Amerindian expansions from current Alaska: the first at 18 thousand years ago (kya) (ending the LGM) [81] and the second at 11 kya (beginning the Holocene) [82]. We investigated several levels of admixture between both populations, including a 100% and 0% contribution of the second population to the final genetic pool. We also simulated other evolutionary scenarios such as ice-sheets derived from the LGM and migration with long-distance dispersal (LDD) events; these are presented in Section 4 and Section 5. The main finding was a simulated genetic gradient with a NW-SE direction throughout the entire continent, which was very similar to the genetic gradient obtained from real data. Importantly, we found that this genetic gradient was invariable with the level of population admixture. We interpreted this gradient as a consequence of IBD caused by the long NW-SE distance of the American continent, and where allele surfing could exist but in a lower extent. That result was for the analysis of the entire continent. Next, we separately analyzed North America to find, for any level of admixture, a gradient with direction NE-SW, perpendicular to the direction of the expansion, that we interpreted as a consequence of allele surfing. This gradient did not fit with the gradients derived from real data. However, as indicated above, we simulated additional evolutionary scenarios (LGM and LDD) to find that the gradient derived from real data in North America can be obtained only if those scenarios are considered (Section 4 and Section 5). The findings suggested that genetic processes such as allele surfing, serial founder events, or IBD, which drive the direction of genetic gradients, can differ among the regions of a landscape.

## 4. Selecting among Alternative Scenarios of Presence and Absence of the Last Glacial Period through Simulated Genetic Gradients

A factor that has been frequently ignored in interpretations of the SE-NW European genetic gradient is the last ice age that occurred at 29–13 kya [83]. During that period, European hunter-gatherer populations probably migrated towards the south through a range contraction, and next, re-expand north to recolonize the areas after the glacial period [84]. Arenas et al. [14] evaluated the influence of the last glacial period on the direction of the European genetic gradient. They performed spatially-explicit computer simulations under the following evolutionary scenarios: (*i*) absence of the last glacial period, (*ii*) presence of the last glacial period through the modeling of a range contraction towards all Southern Europe, followed by a period of time at refugia in all Southern Europe and a posterior re-expansion to recolonize the north and, (*iii*) presence of the last glacial period through the modeling of a range contraction towards only the Iberian Peninsula, followed by a period of time at refugia in only the Iberian Peninsula and a posterior re-expansion to recolonize the north (Figure 2). Note that the scenarios (*ii*) and (*iii*) present a different direction of range re-expansion: a re-expansion with direction S-N in (*ii*) and a re-expansion with direction SW-NE in (*iii*). The range contractions were simulated by a series of progressive contraction events during which demes located in the most northern areas became uninhabitable by setting its carrying capacity to zero [85,86]. In addition, the range contraction was simulated accounting for isotropic and anisotropic migration; the latter was designed to mimic humans who were aware about the glacial period, and that promotes a higher migration towards the south [84]. They found that both range re-expansions produced genetic gradients perpendicular to their direction: the re-expansion S-N led to a genetic gradient with direction E-W and the re-expansion SW-NE led to a genetic gradient with direction NW-SE), but only if the Paleolithic contribution to the final genetic pool was large enough (>80%). It is expected that the last glacial period affects genetic gradients only for large Paleolithic ancestry, because this period occurred during the Paleolithic. The simulated gradients were interpreted as a consequence of allele surfing derived from the range re-expansion, probably because this expansion was recent. Altogether, Arenas et al. [14] found two evolutionary scenarios that fitted better with the real genetic gradients: (*i*) a scenario based on a large Paleolithic ancestry (>95%) and absence of any range contraction, and (*ii*) a scenario with some Paleolithic ancestry that considered a range contraction towards the Iberian Peninsula caused by the last ice period. In contrast, pure Neolithic expansions (without admixture and without genetic signatures from the last ice period) produced genetic gradients that did not fit with the genetic gradients estimated from real data.

Branco et al. [18] studied the influence of ice sheets caused by the last glacial period on the genetic gradients of the entire American continent and North America. It is known that as a consequence of the last glacial period, North America presented two large ice sheets (Laurentide and Cordilleran) that could have affected the entry and settlement of the first modern humans in this continent [87,88]. Concerning the entry to the Americas, two main routes have been proposed (and highly discussed): a coastal route through the North Pacific coastline, and an inland route (ice-free corridor) at the eastern side of the Rocky Mountains [89,90,91]. Indeed, the ice sheets could lead to temporary ice-free refugia in southern regions of North America and posterior expansions to colonize northern regions after melting [92]. In Branco et al. [18], we simulated the colonization of the entire continent and North America considering and ignoring ice sheets derived from the LGM [87]. Following previous works, scenarios with ice sheets were simulated by specifying carrying capacity of the demes covered by ice to zero [85] from 18 kya to 10 kya, a period that considers the duration of ice sheets, frozen grounds, and subsequent inundations [89]. Indeed, the coastal and inland corridors of entry into the Americas were simulated allowing a north to south passage without ice of 1–2 demes (100–200 km) width. At the entire continental level, we found that considering or ignoring the last glacial period does not alter the NW-SE genetic gradient, which was similar to that obtained from real data [6,7,80]. However, in North America, we found that the simulated genetic gradient in absence of the LGM presents a NE-SW direction (which does not fit with the real genetic gradient), while in presence of the LGM it presents a NW-SE direction (similar to the real genetic gradient). We concluded that at the continental level the NW-SE genetic gradient (which was invariable with population admixture and presence/absence of ice sheets) was mainly caused by a strong IBD, probably favored by the long north-south distance of this continent. However, in North America, the ice sheets must be considered to obtain the NW-SE gradient observed from real data. In addition, we also found that migration, including a proportion of long-distance dispersal (LDD) events, favors the simulation of the NW-SE genetic gradient (Section 5.3). Again, these findings suggest that the genetic processes driving the direction of genetic gradients can differ among regions of a landscape.

## 5. Selecting among Alternative Scenarios of Other Evolutionary Processes through Simulated Genetic Gradients

In addition to population admixture and the last glacial period, some other processes were investigated for testing their influence on genetic gradients. In this Section, we also briefly present the application of PC2 and PC3 to identify genetically isolated regions.

### 5.1. Influence of a Paleolithic Expansion from the Iberian Peninsula on the European Genetic Gradient

François et al. [12] investigated a European Paleolithic expansion from the Iberian Peninsula (instead of from the Middle East) followed by a Neolithic expansion from the Middle East. They found that if the simulated Paleolithic ancestry is large (>80%), scenarios with Paleolithic expansion from the Iberian Peninsula lead to genetic gradients similar to those from scenarios with Paleolithic expansion from the Middle East, suggesting that the origin of the Paleolithic expansion does not alter the SE-NW genetic gradient. Because of this, they concluded that the real genetic gradient (SE-NW) was caused by a Paleolithic introgression along the direction of Neolithic expansion instead of just by a Paleolithic range expansion from the Middle East.

### 5.2. Influence of Varying Evolutionary Parameters on Genetic Gradients

Arenas et al. [14] investigated the influence of several evolutionary parameters on European genetic gradients. They found that the genetic gradients were invariable to realistic changes of the ancestral population size, growth rate, and the carrying capacity of Neolithic populations (similar findings were found for American genetic gradients [18]). The only parameter that altered the gradient generated by the Neolithic population was the simulated number of generations. If the number of generations of the simulated Neolithic population is similar to the number of generations of the simulated Paleolithic population (both expansions starting at 40 kya), then the Neolithic population generates a gradient similar to that from the Paleolithic population, supporting the hypothesis that allele surfing could be the cause of the Neolithic genetic gradient (if the expansion is not recent, genetic sectors are lost by homogenization).

### 5.3. Influence of Long-Distance Dispersal on Genetic Gradients

Some studies suggested that the expansion of modern humans throughout the world could present LDD events, for example traveling by boats [93]. Actually, a recent study on the colonization of Eurasia by modern humans found that evolutionary scenarios based on LDD better fitted real data than evolutionary scenarios ignoring LDD [21]. Considering this aspect, in the study of American genetic gradients by Branco et al. [18], we investigated the influence of a proportion of migration through LDD on the genetic gradients. We performed spatially-explicit computer simulations under the LDD model developed by Ray and Excoffier [32], following a LDD distribution estimated from human data [94], a LDD proportion of 5% [21,33], and considering 1,000 km as a maximum distance of dispersal per generation [21]. We found that considering or ignoring LDD does not alter the NW-SE genetic gradient simulated along the entire continent (which is similar to the real genetic gradient [6,7,80]). This again supported the interpretation of strong genetic signatures caused by IBD along the entire American continent. However, in the specific analysis of North America, LDD generated the NW-SE genetic gradient (similar to the real gradient) if there is any genetic contribution from the first (more ancestral) population. This suggested that LDD events that occurred from the first population promoted a homogenization of genetic diversity [33] leading to the gradient that follows the longest geographic distance (an scenario of IBD), while LDD events in only the second expansion would require more time to obtain such homogenization. These findings suggest that LDD events could have occurred in the Americas from the first expansion, explaining the rapid colonization of this continent; this is in agreement with the presence of LDD in previous expansions throughout Eurasia [21].

### 5.4. Evolutionary Information from the Second and Third Principal Components

The first few PCs from a PCA are often used to explore the structure and variance of the data. In the analysis of a genetic sample, the first PC (PC1) map provides a spatial genetic gradient and the following PC maps (especially PC2 and PC3 because the amount of information of the original data is reduced by increasing the PC number) indicates genetically isolated regions. PC2 and PC3 maps were estimated in analyses of European populations [12,14] to highlight Scandinavia and the British Islands as genetically-isolated regions. Concerning the Americas, the inferred PC2 maps showed several regions with genetic isolation: Alaska, the Labrador Peninsula, Central America and Patagonia [18]. All these estimations were in agreement with the findings from real data [6,7,80] and the genetic isolation was mainly explained as a consequence of geographic isolation.

## 6. Conclusions and Future Prospects

Comparisons between simulated and real genetic gradients showed that spatially-explicit computer simulations provide good approximations of real processes and can be used to perform selection among alternative evolutionary scenarios. However, so far all the studies testing alternative scenarios of human evolution through simulated genetic gradients only performed qualitative comparisons with real genetic gradients [12,14,18,78]. In those studies, the fitting between simulated and real gradients was performed just by a visual inspection of their overlapping, and we believe that it is likely that future studies could present situations requiring a quantitative evaluation. As previously indicated, Arenas et al. [14] and Branco et al. [18] performed a high number of computer simulations per studied evolutionary scenario; for each simulated dataset, they estimated a genetic gradient and obtained its direction by connecting the geographical centroids (positive and negative coordinates) to finally compute the median among all the simulated gradients of the evolutionary scenario. We believe that future studies could estimate, in addition to the median, the variance of the simulated gradient for each scenario, and these statistics could be used to perform a quantitative fitting between simulated and real genetic gradients (i.e., with ABC).

Another important aspect in this strategy, as in any analytical strategy based on computer simulations, is that the computer simulations should be as realistic as possible.

The studies discussed in previous sections analyzed genetic gradients of modern humans, ignoring some geographical barriers such as rivers and mountain ranges. We believe that these assumptions may not cause relevant biases when investigating large world regions (as done in such studies), but they could be crucial when investigating small regions. Moreover, the simulations should consider not only the current geographic landscape, but also its evolution from the beginning of the simulated evolutionary history (i.e., accounting for past vegetation maps [95]). Of course, some studies considered the last ice period [14,18], but still, the avalaible resources may vary over time at any region of the landscape, and it was found that a temporal variation of environmental heterogeneity can induce a loss of genetic diversity within demes and increase the population differentiation among demes [96], which we believe could also affect genetic gradients.

Another way to generate more realistic computer simulations is by improving the modeling of human evolution. The aforementioned studies performed computer simulations based on evolutionary parameters (i.e., time and population size at the onset of the expansions, population growth rate, migration rate, LDD proportion, mutation rate, etc) estimated in previous works. However, the real processes were probably more complex, presenting multiple expansion waves and admixtures (e.g., in Europe the Roman and muslim expansions [97,98], or in the Americas, the admixture with non-American populations after the European contact [99,100]), complex demographics, where the population growth rate can vary over time (i.e., caused by population bottlenecks [101]), variation of migration rates over time (which could depend on the lifestyle and technology; for example it was found that Neolithic populations did not expand more rapidly than Paleolithic populations [102], perhaps because of their more sedentary lifestyle, or, as another example, the expansion throughout the Americas was faster than previous expansions throughout other regions [103]), or spatial and temporal selection [104]. Moreover, serial/longitudinal sampling should also be implemented in spatially-explicit computer simulators to analyse the increasing quantity of available ancient genetic data e.g., [105]. In all, the researcher is often forced to identify and apply only those parameters and capabilities implemented in the simulator that could better mimic a desired evolutionary scenario. Hopefully these complex processes will be incorporated into current and future spatially-explicit computer simulators.

In summary, simulated genetic gradients can be useful to perform selections among alternative evolutionary scenarios of modern humans, and we believe that they could also be applied to study other species with similar migration patterns. It is clear that the methods used so far can be improved, especially with more realistic computer simulations (based on high resolution maps and more realistic environmental and evolutionary conditions), and with the application of robust statistical methods for quantitatively evaluating the fitting between simulated and real genetic gradients. We believe that the application of genetic gradients for testing among alternative scenarios will increase in interest and use in the coming years.
